# Phenolic Composition and Antioxidant Activity of Edible Flowers: Insights from Synergistic Effects and Multivariate Analysis

**DOI:** 10.3390/antiox14030282

**Published:** 2025-02-27

**Authors:** Cristiana Breda, Amanda Nascimento, Parkash Meghwar, Hugo Lisboa, Alfredo Aires, Eduardo Rosa, Luís Ferreira, Ana Novo Barros

**Affiliations:** 1 Centre for the Research and Technology of Agro-Environmental and Biological Sciences, CITAB, University de Trás-os-Montes e Alto Douro, UTAD, 5000-801 Vila Real, Portugal; cristianav@utad.pt (C.B.); alfredoa@utad.pt (A.A.); erosa@utad.pt (E.R.); lmf@utad.pt (L.F.); 2 Unidade Académica Engenharia de Alimentos, Universidade Federal Campina Grande, Av. Aprigio Veloso 882, Campina Grande 58429-900, PA, Brazil; amandapriscil@yahoo.com.br (A.N.); hugom.lisboa80@gmail.com (H.L.); 3 Department of Food Science and Technology, University of Karachi, Karachi 75270, Pakistan; kparkash707@gmail.com

**Keywords:** edible flowers, phenolic composition, antioxidant capacity, synergistic effects

## Abstract

The phenolic composition and antioxidant activity of four edible flowers—Orange marigold, yellow marigold, rose geranium, and Rosa de Santa Teresinha—were evaluated to explore their potential as natural antioxidants. Rosa de Santa Teresinha exhibited the highest total phenol content (83.34 ± 2.09 mg GA g^−1^ DW) and ortho-diphenol content (168.91 ± 0.15 mg GA g^−1^ DW), while the marigolds showed significantly lower levels (~17 mg GA g^−1^ DW for total phenols). Antioxidant activity, determined via ABTS, DPPH, and FRAP assays, ranged from 0.11 to 0.96 mmol Trolox g^−1^ DW, with rose geranium and Rosa de Santa Teresinha achieving the highest values. Theoretical antioxidant contributions, calculated based on the identified phenolic compounds, accounted for only a small fraction of the measured activity, with observed values exceeding predictions by factors of 56 to 1416, indicating the presence of synergistic interactions and additional bioactive compounds. Multivariate analyses (PCA and PLS regression) identified luteolin-7-*O*-glucoside and quercetin-3-*O*-galactoside as primary contributors to antioxidant capacity. These results underscore the importance of synergistic effects in edible flowers and highlight their potential as functional ingredients for nutraceutical applications.

## 1. Introduction

Consumption of edible flowers exhibits health benefits because they are a rich source of phytochemicals that minimize the risk of prolonged ailments such as cardiovascular diseases, obesity, and cancer [1]. Globally, flowers have become part of the human diet since ancient times [2] and have become part of edible crops in recent times [1]. Flowers of plant parts that contain phytochemicals have been used for years due to their health benefits and aesthetic properties [3,4].

Functional and nutraceutical foods have gained attention due to positive effects on human wellbeing. Nowadays, edible flowers have increased interest due to their non-toxic nature, food appeal, promoting texture, freshness, and rich phytochemicals (i.e., anthocyanins, flavonoids, phenolic, and carotenoids, etc.) making it good for health and possessing therapeutic properties such as anti-cancer, anti-diabetic, anti-inflammatory, anti-anxiety etc. [1,2,3,4,5]. Ref. [6] reported antioxidants and phytochemicals in more than 200 different edible flowers. Phenolics, carotenoids, flavonoids, and tocols have been reported as the main phyto-chemicals, which results in enhanced antioxidant potential. Several therapeutic properties of edible flowers have been reported by [6], including gastro-protective, geno-protective, hepato-protective, neuro-protective, anti-cancer, antimicrobial, anti-inflammatory, antioxidant, nephro-protective, uricosuric agent, cardio-protective, anti-diabetic, anti-Alzheimer, anti-obesity, and anti-hemolytic. Phytochemicals in edible flowers are found to conjugate with glucose residuals linked to hydroxyl groups, resulting in glycosides [7]. Therefore, edible flowers are known for their antioxidant potential [8]. Ref. [9] discovered the importance of flowers that are edible as food and rich in natural bioactives. Edible flowers have been consumed since ancient times by many cultures (European, Greek, Indian, Chinese, and Roman). They have been used in conventional cuisine, traditional medicine, and as ornaments, mainly due to their appealing and diverse colors [1,2]. Different bioactive compounds were identified in edible flowers, such as phenolic compounds, that are reported to reduce the risk of main chronic diseases [1]. These phytochemicals are responsible for various activities, including anti-obesity, antioxidant, anti-cancer, hypoglycemic, and antimicrobial [10,11,12].

Yellow and orange marigold (*Calendula officinalis* L.) belongs to the family of *Asteraceae*. Marigold has been reported for treatment of some diseases once it presents several bioactivities as antioxidant, anti-inflammatory, anti-diabetic, antibacterial, and anti-fungal, among others [11], which are essentially due to the content of bioactive compounds described in these flowers, namely flavonoids, terpenoids, carotenoids, and quinones [11,12]. *Pelargonium graveolens*, commonly named rose geranium, belongs to the family of *Geraniaceae*. *Pelargonium* species are rich in active compounds, particularly flavonoids, coumarins, gallic and hydroxycinnamic acid derivatives, exhibiting diverse biological activities, such as anti-inflammatory, anti-fungal, and antimicrobial, among others [13,14,15]. Flavonoids, carotenoids, and phenolic acids are described as the main constituents in this flower [16,17,18]. *Rosa damascena* Mill., also known as “Rosa de Santa Teresinha”, belongs to the *Rosaceae* family and has been utilized to heal inflammation, depression, endocrine, and circulation system in ancient times [19]. These edible flowers are rich in vitamins, minerals, carbohydrates, and phenolic compounds [10,20,21] possessing bioactive potential, which can be used in food industries as natural ingredients [22]. Nowadays, synthetic antioxidants substitutes are a topic of debate because of the various serious health issues associated with them [23]. The use of edible flowers as a source of antioxidant compounds can be a good alternative to replacing synthetic food additives. Granato, 2019 [24] studied the chemical composition and antioxidant capacity of marigold flower (*Calendula officinalis* L.) and incorporated the extract of marigold into an organic yogurt. This study demonstrated that *Calendula officinalis* flowers presented high phenolic content and antioxidant capacity. Ref. [25] emphasized that the use of polyphenol-enriched extracts from rose (*Rosa damascena* Mill.) petal by-products in canned strawberries confers an enhancement of antioxidant capacity. The antioxidant capacity and bio-accessibility of phenolic compounds in eight edible flowers (begonia, torenia, cosmos, mini daisy, tagete, clitoria, mini rose, and cravine) were studied by [23]. Also, he explored that cosmos and mini rose are a good source of bio-accessible phenolics with higher antioxidant capacity. For these reasons, the use of edible flowers that are part of the Portuguese flora can represent viable and innovative technological alternatives within the food sector.

This study aims to evaluate the phenolic composition and antioxidant activity of selected edible flowers, investigating the potential synergistic effects between bioactive compounds and their functional applications, allowing the possibility to use edible flowers as a potential substitute for synthetic antioxidants. A comprehensive phytochemical characterization was performed, including the quantification of major phenolic classes (total phenols, *ortho*-diphenols, and flavonoids) and antioxidant capacity assessment using ABTS, DPPH, and FRAP methodologies. Additionally, individual phenolic compounds were identified and quantified by HPLC-DAD. Multivariate analysis was applied to explore correlations between phenolic profiles and antioxidant activity, providing new insights into the complex interactions governing the bioactivity of edible flowers. By elucidating these relationships, this study contributes to the growing body of research on natural antioxidants and their potential applications in food and health industries.

## 2. Materials and Methods

### 2.1. Chemicals

The following chemicals were purchased from Sigma-Aldrich (Steinheim, Germany): methanol, sodium carbonate, sodium molybdate, sodium acetate, gallic acid, catechin, 2,4,6-tris(2-pyridyl)-s-triazine (TPTZ), acetic acid, hydrochloric acid, iron (III) chloride, 2,2′-azino-bis (3-ethylbenzothiazoline-6-sulfonic acid) diammonium salt (ABTS^•+^), 6-Hi-droxy-2,5,7,8-tetra-methylchromone-2-carboxylic acid (Trolox), and 2,2-diphenyl-1-picrylhidrazyl radical (DPPH^•^). Ultrapure water was obtained using a Millipore water purification system. The external commercial standards were obtained from Extrasynthese (Cedex, France). Additionally, aluminum chloride, sodium nitrite, and sodium hydroxide were purchased from Merck (Darmstadt, Germany).

### 2.2. Edible Flowers

The present work was carried out on four edible flowers, namely yellow and orange marigold *Calendula officinalis* L., rose geranium *Pelargonium graveolens*, and “Rosa de Santa Teresinha” *Rosa damascena* Mill, collected in 2024 at Ervas Finas (GPS: 41.24983–7.66407; Vila Real, Portugal). The botanical information of the edible flowers is presented in Table 1. After harvesting, the edible flower petals were transported to the laboratory, frozen at −80 °C and lyophilized. The flower petals (20 flowers) were finely ground into a powder, standardized using 60 Tyler mesh sieves, and then stored under refrigeration conditions (6 ± 2 °C), shielded from light, until the time of analysis.

Each sample was analyzed in triplicate (*n* = 3) for each protocol.

### 2.3. Preparation of Flowers Extracts

For the extraction, 40 mg of dry samples was weighed in triplicate, and 1.5 mL of a methanol/water mixture (70:30, *v*/*v*) was added. The samples were then agitated for 30 min using an orbital shaker (GFL 3005, GEMINI, Apeldoorn, The Netherlands) and subsequently centrifuged at 15,000 rpm for 15 min (Sigma 2-16KL Refrigerated Centrifuges, Sigma Laborzentrifugen, Berlin, Germany). The supernatants were collected in a 5 mL volumetric flask, and this extraction process was repeated three times for each sample. The methanolic extracts of edible flowers were filtered through 0.45 μm PVDF filters (Mil-lex-HV Syringe Filter Unit, Merck Millipore, Bedford, MA, USA).

### 2.4. Phenolic Composition Content

The spectrophotometric methods for determining total phenols, *ortho*-diphenols, and flavonoids were previously reported by [26]. The total phenolic content in edible flower extracts was evaluated using the Folin–Ciocalteu spectrophotometric method, with gallic acid as the standard. Twenty microliters of sample extracts were mixed with 100 microliters of diluted Folin–Ciocalteu reagent and 80 microliters of sodium carbonate (Na_2_CO_3_). The absorbance was measured at 750 nm after incubating the microplate for 30 min at 40 °C in the dark. Results were expressed as milligrams of gallic acid per gram of dry weight (mg GA g^−1^ DW). To assess the *ortho*-diphenol content, 160 microliters of sample extracts was combined with 40 microliters of sodium molybdate solution (Na_2_MoO_4_). The microplate was incubated for 15 min in the dark, and the absorbance was measured at 375 nm, again using gallic acid as the standard. Results were expressed in milligrams of gallic acid per gram of dry weight (mg GA g^−1^ DW). Catechin was used as the standard for determining flavonoid content. Twenty-four microliters of sample extracts and 28 microliters of sodium nitrite (NaNO_2_) were mixed and reacted for 5 min. Subsequently, 28 microliters of aluminum chloride solution (AlCl_3_) was added. After 6 min, 120 microliters of sodium hydroxide (NaOH) was added, and the absorbance was read at 520 nm. The results were expressed in milligrams of catechin per gram of dry weight (mg CAT g^−1^ DW).

### 2.5. Chromatographic Determination of Phenolic Compounds

The phenolic profile of edible flower extracts was evaluated using high-performance liquid chromatography with a diode array detector (HPLC-DAD), as described by [27] The HPLC system included an eluent composed of water with 0.1% trifluoroacetic acid (TFA) (solvent A) and acetonitrile with 0.1% TFA (solvent B). The following linear gradient scheme was applied (t in min; %B): (0, 0%), (5, 0%), (15, 20%), (30, 50%), (45, 100%), (50, 100%), (55, 0%), and (60, 0%). The injection volume for each extract was set at 20 µL, with a flow rate of 1 mL min^−1^. Chromatograms were monitored at 254 and 280 nm for flavan-3-ols and benzoic acids, 320 nm for cinnamic acids, and 370 nm for flavonoids, utilizing a C18 column (250 × 46 mm, 5 µm particle size; ACE HPLC Columns, Advanced Chromatography Technologies Ltd., Aberdeen, Scotland, UK). Individual polyphenols were identified based on UV maximum absorbance bands, peak retention time, UV spectra, and comparison with external commercial standards. In parallel, external standards were prepared (1.0 mg/mL) in a methanol/water (70:30, *v*/*v*) solution and analyzed using HPLC-DAD. The quantity of each polyphenol was determined using the internal standard method.

### 2.6. Antioxidant Capacity

The antioxidant capacity of edible flower extract samples was determined by ABTS^•+^, DPPH^•^, and FRAP spectrophotometric methodologies developed by [26]. The antioxidant capacity was quantified using Trolox as a standard, and the results were expressed in millimoles of Trolox per gram of dry weight (mmol Trolox g^−1^ DW).

#### 2.6.1. ABTS^•+^

The ABTS^•+^ radical was previously generated by mixing 5 mL of ABTS stock solution with 88 μL of potassium persulfate for 12 to 16 h while protecting it from light. For the ABTS working solution, 0.5 mL of the concentrated ABTS solution was combined with 44 mL of sodium acetate buffer, and the absorbance was adjusted to 0.70 ± 0.02 at 734 nm. A mixture of 188 μL of the ABTS working solution and 12 μL of the sample was incubated for 30 min in the dark and measured at 734 nm.

#### 2.6.2. DPPH

The DPPH solution was prepared by mixing 35 mg of Trolox with 10 mL of methanol, presenting an absorbance of 1.000 at 520 nm. Afterwards, 188 μL of DPPH and 10 μL of the sample were reacted for 30 min, protected from light, and the absorbance was read at 520 nm.

#### 2.6.3. FRAP

The FRAP working solution was performed by mixing 10 volumes of acetate buffer with 1 volume of TPTZ and 1 volume of ferric chloride. The FRAP solution was maintained for 10 min at 37 °C. Then, 280 μL of FRAP working solution and 20 μL of sample were added, and the mixture was incubated for 30 min at 37 °C, protected from light, before the absorbance at 593 nm was read.

### 2.7. Mechanistic Antioxidant Contribution Calculations

Theoretical antioxidant contributions of individual phenolic compounds were calculated based on their concentrations determined by high-performance liquid chromatography (HPLC) and literature-derived Trolox equivalent antioxidant capacity (TEAC) values and presented in Table S1. The concentrations of each compound were converted to a molar basis (mmol g^−1^ DW) using their respective molecular weights. Theoretical contributions of each compound to ABTS, DPPH, and FRAP antioxidant activity were calculated using Equation (1).

(1) $$Contribution\left(\frac{mmol\;Trolox}{g}\;\right)=\frac{Average\;Concetration\;(\frac{\mu g}{g})\;}{Mw\;(\frac{g}{mol})}\times TEAC\times 10^{-3}$$

The total theoretical antioxidant activity for each flower extract was obtained by summing the individual contributions of all identified phenolic compounds for each assay (ABTS, DPPH, FRAP). The calculated theoretical contributions were compared to experimentally measured antioxidant activities obtained through ABTS, DPPH, and FRAP assays (Section 2.5). The percentage of antioxidant activity explained by the identified compounds was determined using Equation (2).

(2) $$Explained\;\left(\%\right)=\frac{Contribution}{Measured\;activity\;}\times 100$$

This methodology allowed for quantifying individual phenolic contributions to overall antioxidant activity and provided insights into potential gaps between measured and theoretical values, highlighting the roles of unidentified compounds or synergistic effects. The synergy factor assesses the extent to which the interaction among compounds enhances antioxidant activity beyond additive effects. It is typically calculated as Equation (3).

(3) $$Synergy\;Factor=\frac{Measured\;activity}{Sum\;of\;Theoretical\;Contributions\;}$$

### 2.8. Multivariate Statistical Analysis

Principal component analysis (PCA) and partial least squares (PLS) regression were conducted to identify key bioactive compounds influencing antioxidant activity. Concentrations of phenolic compounds (X-matrix) and antioxidant assay results (ABTS, DPPH, FRAP; Y-matrix) were standardized (mean = 0, variance = 1) before analysis. PCA was performed to reduce data dimensionality and visualize sample clustering. PLS regression modeled the relationships between phenolic profiles and antioxidant capacity, with variable importance in projection (VIP) scores greater than 1.0 indicating significant predictors. Cross-validation (k-fold, k = 5) was utilized to evaluate model robustness.

### 2.9. Statistical Analysis

All experimental measurements were conducted in triplicate, and results are presented as the mean ± standard deviation. Statistical analyses were carried out using one-way analysis of variance (ANOVA), followed by Tukey’s post hoc test to identify significant differences between groups. The normality and homoscedasticity of the data were evaluated using the Shapiro–Wilk and Levene’s tests, respectively. Differences were deemed statistically significant at *p* < 0.05. Statistical analyses were performed using GraphPad Prism (version 10.4.1).

## 3. Results and Discussion

### 3.1. Bioactive Compound Profile of Edible Flowers

The analysis of the phenolic composition revealed significant differences among the edible flower species studied. Rosa de Santa Teresinha exhibited the greatest diversity and concentration of flavonoids, especially quercetin-3-*O*-galactoside and luteolin-7-*O*-glucoside, compounds associated with high antioxidant capacity. Rose geranium showed an intermediate profile, standing out for its levels of catechin and epicatechin, which also play a fundamental role in antioxidant activity. On the other hand, the Marigold varieties (both orange and yellow calendula) distinguished themselves with an abundance of phenolic acids, such as caffeic acid and chlorogenic acid, which contribute to free radical scavenging. These results indicate that differences in phenolic composition directly impact the antioxidant activity of the species, with flavonoid-rich flowers exhibiting greater antioxidant potential, while varieties predominantly composed of phenolic acids may act in a complementary manner in nutraceutical and food applications.

In fact, the HPLC analysis (Table 2) revealed significant differences (*p* < 0.05) in the concentrations of bioactive compounds among the evaluated edible flowers. Orange marigold showed the highest levels of phenolic acids, while Rosa de Santa Teresinha displayed a greater diversity of flavonoids, anthocyanins, and catechins.

Phenolic acids, recognized for their role in reducing oxidative stress, were found in varying concentrations among the species analyzed. Orange marigolds displayed the highest levels of caffeic acid (101.10 ± 9.07 µg/g), followed by yellow marigolds (71.61 ± 1.53 µg/g). Caffeic acid is noted for its potent antioxidant and anti-inflammatory properties, including the modulation of NF-κB transcriptional pathways [28,29]. Chlorogenic acid, also abundant in these species, has been linked to improved glycemic metabolism and cardiovascular protection [30,31]. In contrast, rose geranium showed significantly lower levels of phenolic acids, indicating a reduced antioxidant potential.

**Table 2:** Phenolic composition (µg/g) of edible flower extracts. Concentrations of individual phenolic compounds identified in yellow marigold, orange marigold, rose geranium, and Rosa de Santa Teresinha. Values are presented as mean ± standard deviation.

Compounds	Yellow Maringold (μg/g)	Orange Maringold (μg/g)	Rose Germanium (μg/g)	Rosa Santa Teresinha (μg/g)
Chlorogenic acid	34.10 ± 2.27 a	37.15 ± 2.7 a	14.11 ± 1.88 b	ND
4-Caffeoylquinic acid	4.90 ± 0.42 a	2.77 ± 0.1 b	ND	ND
Caffeic acid	71.61 ± 1.52 b	101.10 ± 9.1 a	ND	ND
*m*-cumaric acid	41.04 ± 1.99 b	49.49 ± 1.5 a	10.53 ± 0.43 c	ND
*p*-cumaric acid	17.31 ± 1.44 b	24.72 ± 1.9 a	6.53 ± 0.42 c	24.32 ± 0.42 a
Diosmetine	12.08 ± 0.63	ND	ND	ND
Diosmetin 6,8-*O*-glucoside	ND	16.03 ± 1.4 a	ND	ND
Quercetin-3-*O*-rutinoside	36.61 ± 1.17 b	45.03 ± 0.69 a	5.64 ± 0.44 c	20.59 ± 3.5 d
Quercetin-3-*O*-galactoside	3.85 ±0.53 c	3.09 ± 0.53 c	14.89 ± 1.91 b	51.51 ± 4.46 a
Quercetin-3-*O*-glucoside	7.01 ± 1.46 b	6.92 ± 1.05 b	9.63 ± 0.82 a	ND
Quercetin-3-*O*-xyloside	18.68 ± 1.25 b	19.41 ± 2.49 ab	22.47 ± 1.2 a	ND
Quercetin-3-*O*-pentoside	4.29 ± 3.73 b	ND	16.53 ± 2.4 a	ND
Quercetin-3-*O*-rhamnoside	ND	ND	14.24 ± 0.57	ND
Luteolin-3-*O*-glucoside	108.33 ± 7.2 a	105.19 ± 2.03 a	ND	ND
Luteolin-glycoside I	ND	ND	ND	46.42 ± 4.91
Luteolin-glycoside II	ND	ND	ND	23.37 ± 2.1
Luteolin-7-*O*-glucoside	ND	ND	ND	144.13 ± 12.5
Luteolin-glycoside III	ND	ND	ND	31.63 ± 2.8
Luteolin-glycoside IV	ND	ND	ND	65.12 ± 5.2
Luteolin-glycoside V	ND	ND	ND	31.28 ± 2.7
Cyanidin-3-*O*-glucoside	ND	ND	ND	6.37 ± 0.16
Delphinidin	ND	ND	ND	ND
Catechin	ND	ND	11.22 ± 2.3	ND
Eriodictyol	ND	ND	14.64 ± 0.1	ND
Epigallocatechin gallate	ND	ND	10.15 ± 0.14	ND
Epicatechin	ND	ND	6.49 ± 0.58	ND
Galocatechin gallate	ND	ND	3.99 ± 0.35	ND
Epicatechin gallate	ND	ND	2.61 ± 0.24	ND
Catechin gallate	ND	ND	3.73 ± 0.35	ND

Values are expressed as mean ± SD (*n* = 3). Within each row, means followed by different letters are significantly different (*p* < 0.05) according to one-way ANOVA with Tukey’s post hoc test. Compared to the marigolds and Rosa de Santa Teresinha. Notably, Rosa de Santa Teresinha distinguished itself with its high concentration of *p*-coumaric acid (24.33 ± 0.43 µg/g), a compound known for its antimicrobial activity and its role in maintaining cellular integrity [32] These findings are consistent with previous reports that established a correlation between conjugated phenolic acids and enhanced antioxidant performance in assays such as ABTS and FRAP [27].

Flavonoids displayed unique chemical profiles across the species studied. Rosa de Santa Teresinha had the highest concentration of quercetin-3-*O*-galactoside (77.27 ± 0.58 µg/g), a compound recognized for its strong antioxidant and anti-inflammatory properties [33]. In contrast, orange marigold was particularly abundant in quercetin-3-*O*-rutinoside (45.04 ± 0.69 µg/g), a flavonoid that aids in stabilizing cellular membranes and regulating apoptosis [34]. Yellow marigold was the only species containing diosmetin, which is important for immune regulation and protection against cellular aging [35] (. Rose geranium showed the lowest flavonoid concentrations, indicating limited functional diversity. The presence of these compounds is especially relevant given their well-documented roles in reducing the risk of cardiovascular and inflammatory diseases [33,34].

Anthocyanins were found exclusively in Rosa de Santa Teresinha, which reinforces its distinctive antioxidant profile. This species contained cyanidin-3-*O*-glucoside at a concentration of 6.37 ± 0.16 µg/g, aligning with studies that connect anthocyanins to neuroprotection and a lowered risk of neurodegenerative diseases such as Alzheimer’s and Parkinson’s [36,37]. The absence of anthocyanins in marigold species and rose geranium limits their antioxidant potential compared to Rosa de Santa Teresinha. The intense pigmentation observed in this species may indicate its high anthocyanin content, which corresponds with findings from [19] regarding Rosa damascena, a related species rich in anthocyanins and hydroxycinnamic acids. These compounds are well recognized for their capacity to modulate cellular signaling pathways and offer vascular protection [36].

Catechins were found exclusively in Rosa de Santa Teresinha, emphasizing its potential contributions to cardiovascular health and energy metabolism regulation. This species displayed notable concentrations of catechin (11.22 ± 2.29 µg/g) and epigallocatechin gallate (EGCG, 10.15 ± 1.35 µg/g), two compounds recognized for their effectiveness in lowering LDL cholesterol and enhancing endothelial function [38,39]. The lack of catechins in other species limits their potential use as antioxidant-rich foods or nutraceuticals. Research indicates that these molecules may have a protective role against metabolic disorders, further highlighting the exceptional bioactive potential of Rosa de Santa Teresinha.

In summary, the observed variations in phenolic content among the edible flowers not only illustrate distinct chemical profiles but also have important functional implications. For instance, the high concentration of flavonoids in Rosa de Santa Teresinha is closely linked to its robust antioxidant potential, suggesting that this species could play a significant role in mitigating oxidative stress and associated inflammatory processes. In contrast, the predominance of phenolic acids in marigold varieties indicates a strong capacity for free radical scavenging, which may contribute to protective effects against cellular damage and support metabolic health. Additionally, the unique presence of anthocyanins and catechins in Rosa de Santa Teresinha highlights its potential in modulating cellular signaling pathways and enhancing cardiovascular and neuroprotective functions. Together, these functional interpretations emphasize that the compositional differences are not merely numerical but also translate into meaningful biological activities, underscoring the promise of these edible flowers in nutraceutical and functional food applications.

### 3.2. Phenolic Composition

**Figure 1:**
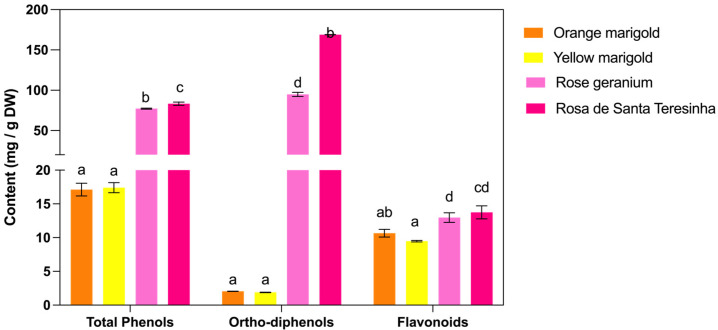
Total phenols, ortho-diphenols, and flavonoids in edible flower extracts. Bars represent the concentrations of total phenols (mg GA g^−1^ DW), *ortho*-diphenols (mg GA g^−1^ DW), and flavonoids (mg CAT g^−1^ DW) measured in yellow marigold, orange marigold, rose geranium, and Rosa de Santa Teresinha. Error bars indicate the standard deviation of triplicate measurements. Different letters above bars indicate significant differences (*p* < 0.05) among the flowers within each compound class.

Figure 1 shows the total phenolic composition of the characterized edible flowers. The results demonstrate significant variability in phenolic composition, indicating differences in their phytochemical profiles and potential bioactive properties. Rosa de Santa Teresinha exhibited the highest total phenol content (83.34 ± 2.09 mg GA g^−1^ DW), closely followed by rose geranium (77.19 ± 0.65 mg GA g^−1^ DW), while orange and yellow marigolds displayed considerably lower values (approximately 17 mg GA g^−1^ DW, *p* > 0.05). Similar findings have been reported by [34,36,37,38,39,40], who noted that variations in total phenolic content are often associated with differences in plant species and their metabolic pathways. These results suggest that marigold species accumulate fewer phenolics than rose varieties, likely due to differences in the regulation of polyphenol biosynthesis [20,21,22].

A similar trend was observed in ortho-diphenol content. Rosa de Santa Teresinha contained significantly higher levels (168.91 ± 0.15 mg GA g^−1^ DW) than all other flowers, while rose geranium also exhibited elevated concentrations (94.89 ± 2.56 mg GA g^−1^ DW). In contrast, the marigold varieties had very low levels (approximately 2 mg GA g^−1^ DW, *p* > 0.05). *Ortho*-diphenols are known for their strong radical-scavenging properties, and higher levels are correlated with enhanced antioxidant performance [41]. These results support the notion that Rosa de Santa Teresinha and rose geranium are richer in ortho-diphenol-containing compounds—such as certain flavonols, anthocyanins, and phenolic acids—which likely contribute to their superior antioxidant properties.

The distribution pattern of flavonoid content differed from that of total phenols and ortho-diphenols. Yellow marigold exhibited the lowest flavonoid concentration (9.47 ± 0.11 mg CAT g^−1^ DW), which was significantly lower than the levels observed in rose geranium (12.97 ± 0.71 mg CAT g^−1^ DW) and Rosa de Santa Teresinha (13.75 ± 0.97 mg CAT g^−1^ DW). Orange marigold displayed an intermediate flavonoid content (10.66 ± 0.57 mg CAT g^−1^ DW), with no significant differences from the other samples. These variations in flavonoid levels align with previous studies indicating that rose extracts generally possess higher concentrations of flavonoid compounds than marigold extracts [42,43]. The elevated flavonoid levels in rose geranium and Rosa de Santa Teresinha may be attributed to their higher content of flavonol and flavone derivatives, which are linked to enhanced antioxidant and anti-inflammatory activities [33,34]. In contrast, the lower values seen in marigolds might signify a phytochemical profile that favors other classes of antioxidants, such as carotenoids [44].

Overall, these results confirm that Rosa de Santa Teresinha is the richest source of phenolic compounds, particularly ortho-diphenols, while rose geranium stands out for its higher flavonoid content. The marigold species displayed a more modest phenolic profile, with yellow marigold showing the lowest levels of flavonoids. Such differences in phytochemical composition—likely influenced by botanical origin, metabolic pathways, and environmental factors—are well documented in the literature [20,21,22]. Given the established health benefits of phenolics [34,36,37,38,39,40] these findings underscore the potential of Rosa de Santa Teresinha and rose geranium as valuable sources of bioactive compounds for functional food and nutraceutical applications.

### 3.3. Antioxidant Capacity of Edible Flowers and Mechanistic Estimation

The antioxidant capacity of the edible flower extracts was evaluated using ABTS^•+^, DPPH^•^, and FRAP assays, and the experimental results are presented in Table 3. In parallel, theoretical antioxidant contributions were estimated from the HPLC-identified phenolic composition, with detailed calculations provided in Supplementary Table S2. This dual approach quantitatively measures each flower’s overall radical-scavenging potential while offering mechanistic insight into the primary molecular contributors and their modes of action [45,46].

Among the samples, Rosa de Santa Teresinha and rose geranium exhibited the highest antioxidant capacities across all assays. Specifically, Rosa de Santa Teresinha demonstrated the highest activity in the ABTS assay (0.93 ± 0.02 mmol Trolox g^−1^ DW), whereas rose geranium showed superior performance in the DPPH (0.96 ± 0.00 mmol Trolox g^−1^ DW) and FRAP (0.85 ± 0.02 mmol Trolox g^−1^ DW) assays. In contrast, the Tagetes species (orange and yellow marigolds) consistently exhibited significantly lower values. These experimental results align with previous reports that link high flavonoid and phenolic acid contents—especially flavonols like quercetin derivatives and hydroxycinnamic acids such as caffeic acid—with strong radical-scavenging activity [47,48]. Furthermore, the strong antioxidant performance observed in Rosa de Santa Teresinha and rose geranium suggests that their high concentrations of flavonoid glycosides contribute not only through direct hydrogen or electron donation but also via mechanisms such as metal chelation [44].

The theoretical antioxidant potential, derived from the measured concentrations of individual phenolic compounds and their literature-reported Trolox equivalent antioxidant capacity (TEAC) values, revealed significant inter-species variation. In the marigold species, antioxidant activity was primarily driven by hydroxycinnamic acids—specifically, caffeic and chlorogenic acids—which neutralize free radicals through hydrogen atom donation, a mechanism well documented in the literature [49,50]. The higher theoretical contribution from these acids suggests that a given marigold species may contain a relatively greater proportion of bioavailable phenolic acids, with caffeic acid playing a particularly crucial role in radical scavenging. In contrast, the theoretical antioxidant contributions of rose geranium were mainly connected to flavonol derivatives such as quercetin-3-*O*-galactoside, quercetin-3-*O*-rutinoside, and quercetin-3-*O*-xyloside. Although these compounds are potent antioxidants, their glycosylated forms may diminish reactivity compared to hydroxycinnamic acids by influencing bioavailability and redox cycling [51,52]. Their combined presence suggests additional roles in metal chelation and the inhibition of lipid peroxidation [53]. The theoretical calculations for Rosa de Santa Teresinha identified luteolin glycosides—particularly luteolin-7-*O*-glucoside—as major contributors, alongside quercetin derivatives. Luteolin glycosides are well recognized for their high efficiency in radical scavenging, metal chelation, and stabilization of oxidative damage [51,52]. Moreover, the exclusive presence of cyanidin-3-*O*-glucoside, an anthocyanin found only in Rosa de Santa Teresinha, further supports its potential role in redox modulation and lipid membrane protection [54].

Overall, the mechanistic evaluation confirms that the phenolic profile is a key determinant of the radical-scavenging properties of edible flowers. Flowers rich in hydroxycinnamic acids, like marigold species, primarily act through hydrogen donation mechanisms. In contrast, those abundant in flavonols and flavones, such as rose geranium and Rosa de Santa Teresinha, rely more on electron transfer and metal chelation. Despite these detailed estimations, a notable discrepancy was observed between the theoretical antioxidant contributions and the experimentally measured activities. For instance, in Rosa de Santa Teresinha, the identified phenolics accounted for only 0.36% of ABTS, 0.31% of DPPH, and 0.38% of FRAP activity. Similar low percentages were noted for rose geranium and marigold species, indicating that a significant portion of the total antioxidant capacity is derived from unidentified bioactive compounds and synergistic interactions among extract constituents. Unidentified antioxidants—such as polymeric tannins, additional anthocyanins, carotenoids, and other non-phenolic compounds that may not be captured by standard HPLC methods—could substantially contribute to the overall activity [41,42,43,44,45,46,47,48,49,50,51,52,53,54,55]. Furthermore, synergistic interactions between compounds enhance radical-scavenging activity beyond the sum of individual contributions [56], and methodological differences between the controlled conditions for TEAC determinations and those used in the assays likely contribute to this underestimation [57]. These combined factors

### 3.4. Multivariate Analysis of Phenolic Composition and Antioxidant Activity

Multivariate statistical analyses—including principal component analysis (PCA) and partial least squares regression (PLSR)—were employed to investigate patterns in phenolic composition and identify the key compounds linked to antioxidant activity. These chemometric methods enable a thorough evaluation of the phytochemical diversity among edible flowers and offer mechanistic insights into the relationship between individual phenolics and the overall antioxidant capacity [58,59].

#### 3.4.1. Principal Component Analysis (PCA) of Phenolic Composition

**Figure 2:**
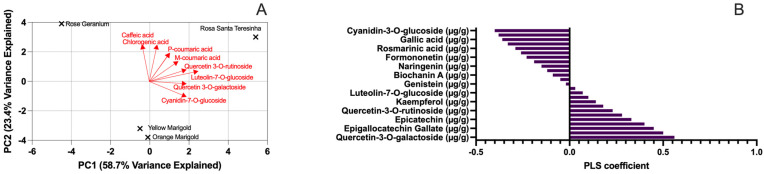
Multivariate analysis of phenolic composition and antioxidant contributions in edible flowers. (**A**) PCA biplot illustrating the phenolic composition of edible flowers. PC1 explains 58.7% of the variance, driven by luteolin and quercetin derivatives, while PC2 explains 23.4%, associated with hydroxycinnamic acids and catechins. Sample clustering shows Rosa Santa Teresinha’s dominance in luteolin glycosides and rose geranium’s mixed phenolic profile. (**B**) PLS regression coefficients for total phenols, highlighting the primary phenolic compounds contributing to antioxidant capacity. Luteolin-7-*O*-glucoside and quercetin-3-*O*-galactoside exhibit the strongest positive contributions, while compounds like chlorogenic acid and caffeic acid display moderate effects.

PCA was conducted to identify the primary sources of variation in the phenolic composition of the analyzed edible flowers. The first two principal components (PC1 and PC2) accounted for 81.0% of the total variance, with PC1 explaining 58.3% and PC2 explaining 22.7% (see Table S1, Supplementary Material). The loadings show that luteolin glycosides and quercetin derivatives were the primary contributors to PC1, while hydroxycinnamic acids (e.g., caffeic acid and chlorogenic acid) were predominant in PC2. The PCA biplot (Figure 2) illustrates that Rosa de Santa Teresinha clusters with high PC1 scores—indicating its richness in luteolin glycosides (e.g., luteolin-7-*O*-glucoside, luteolin-glycoside I–V) and quercetin derivatives (e.g., quercetin-3-*O*-galactoside, quercetin-3-*O*-rutinoside). In contrast, rose geranium shows a more intermediate profile, while yellow and orange marigolds cluster together, suggesting that hydroxycinnamic acids, rather than flavonoids, are more abundant in their phenolic profiles. These findings are consistent with previous food chemistry research that showed how phenolic profile variations affect sample clustering and functional diversity [60,61]. 

The PCA biplot displays the clustering of edible flower samples based on standardized data of phenolic compound concentrations (X-matrix) and antioxidant assay results (ABTS, DPPH, FRAP; Y-matrix). The percentages shown on each axis (e.g., 52.31% on PC1 and 21.67% on PC2) represent the proportion of the total variance explained by each principal component. This means that PC1 accounts for 52.31% of the variability in the dataset, while PC2 explains 21.67%, together capturing approximately 74% of the overall variance. Such high explained variance confirms that these two components effectively summarize the most significant differences among the samples. In addition, the loading vectors (arrows) indicate the direction and magnitude of the influence exerted by individual phenolic compounds on the data structure, highlighting which compounds are key contributors to the observed clustering. Complementing the PCA, partial least squares (PLS) regression was applied to model the relationship between phenolic profiles and antioxidant activity, with variable importance in projection (VIP) scores greater than 1.0 indicating significant predictors, as confirmed by a 5-fold cross-validation. This integrated approach not only visualizes sample clustering but also provides functional insights into the bioactive compounds driving antioxidant performance.

#### 3.4.2. Partial Least Squares Regression (PLSR) for Predicting Antioxidant Activity

PLSR was utilized to identify phenolic compounds that most effectively predict antioxidant activity, as measured using the ABTS, DPPH, and FRAP assays. The PLS model demonstrated strong predictive capability, accounting for 85% of the ABTS variance, 81% of the DPPH variance, and 78% of the FRAP variance (see Table S2, Supplementary Material). Variable importance in projection (VIP) scores indicated that luteolin-7-*O*-glucoside (VIP = 2.5), quercetin-3-*O*-galactoside (VIP = 2.1), and luteolin-glycoside IV (VIP = 2.4) were the most significant predictors across the assays. Secondary contributors included caffeic acid (VIP = 1.8) and p-coumaric acid (VIP = 1.5), compounds known for their radical-scavenging properties [62,63]. The standardized regression coefficients (see Table S3, Supplementary Material) further confirmed that flavonoid glycosides—especially those based on luteolin and quercetin—are strongly correlated with antioxidant potential. Notably, the high VIP scores for caffeic acid and p-coumaric acid in marigold species (see Table S6, Supplementary Material) highlight that hydroxycinnamic acids significantly contribute to radical-scavenging activity, despite their lower concentration compared to flavonoids.

#### 3.4.3. Insights from Multivariate Analysis

The PCA and PLSR results confirm that flavonoid glycosides (such as luteolin and quercetin derivatives) and hydroxycinnamic acids are key determinants of antioxidant activity in edible flowers. The PCA clustering clearly distinguishes Rosa de Santa Teresinha, rose geranium, and marigold species based on their phenolic profiles, while the PLSR emphasizes the varying contributions of individual compounds to antioxidant capacity. These findings reinforce that the high antioxidant activity of Rosa de Santa Teresinha primarily stems from its rich flavonoid content, particularly luteolin glycosides, while marigolds obtain most of their antioxidant potential from hydroxycinnamic acids—even if at a lower overall level. The predictive power of the PLSR model suggests that incorporating broader metabolomic analysis (including polymeric tannins and carotenoids) may further clarify the antioxidant mechanisms in these edible flowers [64].

### 3.5. Mechanistic Insights into the Antioxidant Action of Edible Flowers

This section examines how the identified phenolic compounds in the four edible flower species contribute to scavenging reactive oxygen species (ROS), reactive nitrogen species (RNS), and reactive sulfur species (RSS) and modulate antioxidant enzyme systems.

#### 3.5.1. ROS, RNS, and RSS Scavenging Mechanisms in Edible Flowers

The phenolic acids, flavonoids, and anthocyanins present in the flower extracts show significant antioxidant potential through various pathways. The HPLC analysis indicated that orange marigolds and yellow marigolds contain high concentrations of caffeic acid, chlorogenic acid, and p-coumaric acid, which serve as direct radical scavengers through hydrogen donation mechanisms [49,50]. Caffeic acid (101.1 µg/g in orange marigold, 71.6 µg/g in yellow marigold) plays a critical role in neutralizing hydroxyl radicals (^•^OH) and superoxide anions (O_2_^•−^) due to its ortho-dihydroxy structure, allowing electron transfer and stabilizing reactive radicals. This function is particularly essential in inhibiting lipid peroxidation, thereby preventing oxidative damage to cellular membranes. Chlorogenic acid (37.2 µg/g in orange marigold, 34.1 µg/g in yellow marigold) offers dual protective effects by chelating transition metals such as iron (Fe^2+^), thus preventing Fenton reactions that produce hydroxyl radicals, and by scavenging peroxynitrite (ONOO^−^), which is crucial in nitrosative stress. p-coumaric acid (24.3 µg/g in Rosa de Santa Teresinha, 6.5 µg/g in rose geranium) effectively neutralizes nitrogen dioxide radicals (NO_2_^•^), reducing protein nitration and inflammatory responses. The data suggest that marigold species function primarily as ROS scavengers due to their high hydroxycinnamic acid content. In contrast, Rosa de Santa Teresinha exhibits greater RNS scavenging potential, attributed to its higher levels of p-coumaric acid.

Flavonoids play a critical role in antioxidant defense by employing various mechanisms, including electron transfer, metal chelation, and modulation of oxidative stress pathways. The high levels of quercetin derivatives found in Rosa de Santa Teresinha and rose geranium significantly enhance their antioxidant properties. Quercetin-3-*O*-galactoside (51.5 µg/g in Rosa de Santa Teresinha and 14.9 µg/g in rose geranium) has been shown to scavenge superoxide anions and hydroxyl radicals, as well as chelate Fe^2+^ and Cu^2+^ ions, helping to prevent oxidative DNA damage [51]. Luteolin-7-*O*-glucoside (144.1 µg/g in Rosa de Santa Teresinha) is particularly effective at quenching peroxyl radicals (ROO^•^), inhibiting lipid peroxidation, and reducing nitrosative stress by downregulating iNOS (inducible nitric oxide synthase) [52]. Diosmetin (12.1 µg/g in yellow marigold) also contributes to antioxidant defense by scavenging peroxyl radicals and downregulating nuclear factor kappa B (NF-κB), a key transcription factor involved in inflammatory responses [53]. These findings highlight that Rosa de Santa Teresinha and rose geranium exhibit strong antioxidant defense through flavonoid-mediated mechanisms, positioning them as promising candidates for functional food applications.

Anthocyanins are particularly significant in relation to Rosa de Santa Teresinha, the only species in this study that contains cyanidin-3-*O*-glucoside (6.37 µg/g). Anthocyanins contribute to redox homeostasis by scavenging singlet oxygen (^1^O_2_) and peroxyl radicals (ROO^•^). Moreover, they demonstrate neuroprotective properties by preventing RNS-induced apoptosis, underscoring their potential role in reducing oxidative damage linked to neurodegenerative disorders. The high antioxidant capacity of Rosa de Santa Teresinha, as shown in the ABTS assay (0.93 mmol Trolox g^−1^ DW), correlates well with its anthocyanin content [52].

#### 3.5.2. Modulation of Antioxidant Enzyme Systems

In addition to their direct radical scavenging activities, literature reports indicate that the phenolic compounds present in edible flowers have the potential to modulate the activity of key antioxidant enzymes such as superoxide dismutase (SOD), catalase (CAT), and glutathione peroxidase (GPx). Although our study did not directly measure changes in enzyme activities, the high levels of specific phenolics in our samples suggest possible roles in enzyme modulation as described in previous research.

For instance, flavonoids such as quercetin-3-*O*-galactoside (51.5 µg/g in Rosa de Santa Teresinha) and luteolin (144.1 µg/g in Rosa de Santa Teresinha) have been reported to enhance SOD expression via activation of the Nrf2 (nuclear factor erythroid 2-related factor 2) signaling pathway [26]. This activation promotes the dismutation of superoxide anions (O_2_^•−^) into hydrogen peroxide (H_2_O_2_), thereby reducing oxidative stress and contributing to cellular homeostasis.

Similarly, hydroxycinnamic acids—specifically caffeic acid and chlorogenic acid, which are found at high levels in orange marigold (101.1 µg/g) and in both marigold species—have been associated with the upregulation of catalase (CAT). CAT facilitates the conversion of hydrogen peroxide into water and oxygen (2H_2_O_2_ → 2H_2_O + O_2_; see Equation (4)), suggesting that marigold extracts might help mitigate hydrogen peroxide-induced oxidative damage, a feature that could be valuable in food preservation.

Furthermore, flavonoids like quercetin and luteolin, abundant in Rosa de Santa Teresinha and rose geranium, have been linked to enhanced glutathione peroxidase (GPx) activity. GPx is critical for neutralizing lipid hydroperoxides and maintaining glutathione homeostasis by converting oxidized glutathione (GSSG) back to its reduced form (GSH). (GPx reaction: 2GSH + H_2_O_2_ → GSSG + 2H_2_O; see Equation (5)) [54].

While our data do not include direct measurements of enzyme activities, these literature-based insights support the hypothesis that the phenolic profiles of edible flowers can contribute to the modulation of antioxidant enzyme systems.

#### 3.5.3. Linking Antioxidant Activity to Biological Function

The integrated analysis of phenolic composition and antioxidant activity suggests that different edible flower species may contribute to cellular antioxidant defenses through multiple mechanisms. Marigold species, which are rich in hydroxycinnamic acids such as caffeic acid, chlorogenic acid, and p-coumaric acid, likely act as potent scavengers of hydroxyl radicals and peroxynitrite, supporting their potential as natural food antioxidants [65]. In contrast, Rosa de Santa Teresinha, renowned for its high flavonoid and anthocyanin content, has been reported in the literature to enhance the activities of antioxidant enzymes (SOD, CAT, and GPx), thereby offering robust cellular protection. Similarly, rose geranium, distinguished by its flavonoid-rich profile, may bolster antioxidant defenses through mechanisms such as metal chelation and the inhibition of inflammatory pathways [51].

It is important to note that while our study did not directly measure changes in enzyme activities, the notable discrepancy between theoretical and experimentally measured antioxidant activities indicates strong synergistic interactions among the phenolic compounds. This synergy emphasizes the potential superiority of whole-flower extracts over isolated compounds for achieving optimal efficacy. These combined findings advocate further investigation into the use of edible flowers in developing functional foods and nutraceutical formulations to combat oxidative stress and inflammation-related disorders [52].

## 4. Conclusions

This study demonstrates a complex interplay between the phenolic composition of edible flowers and their antioxidant activities, offering fresh insights into their bioactive potential. Our findings reveal that the overall antioxidant capacity observed in these flowers far exceeds the sum of the contributions predicted by individual phenolic compounds. This discrepancy highlights the critical role of synergistic interactions and suggests that unidentified bioactive constituents also significantly contribute to the antioxidant effect.

Notably, Rosa de Santa Teresinha and rose geranium exhibit superior antioxidant activities, a result of their higher concentrations of flavonoids and ortho-diphenols. These species show great promise for applications in functional foods and nutraceuticals, where a high antioxidant capacity is highly desirable. In contrast, marigold species, with their moderate antioxidant profiles primarily driven by hydroxycinnamic acids, may serve as complementary ingredients in formulations with less stringent antioxidant demands.

Furthermore, our multivariate analysis (PCA and PLS regression) provided deeper insights into the phenolic drivers of antioxidant activity, particularly underscoring the significance of luteolin glycosides and quercetin derivatives. By integrating robust statistical modeling with experimental data, our study advances understanding beyond traditional additive models, offering a more nuanced view of the relationship between phenolic composition and bioactivity.

Overall, these results reinforce the value of edible flowers as diverse and complex sources of natural antioxidants. They emphasize the need for holistic approaches that account for both identified and unidentified synergistic effects, paving the way for future research to optimize the use of these bioactive-rich matrices in health-promoting applications and to further refine methodologies for evaluating natural bioactive systems.

## Figures and Tables

**Table 1:** Botanical information of edible flowers.

Common Name	Scientific Name
Yellow marigold	*Calendula officinalis* L.
Orange marigold	*Calendula officinalis* L.
Rose geranium	*Pelargonium graveolens*
“Rosa de Santa Teresinha”	*Rosa damascena* Mill.

**Table 3:** Comparison of theoretical and measured antioxidant activities (ABTS, DPPH, FRAP) and synergy factors for edible flowers.

Edible Flowers	Units	Orange Marigold	Yellow Marigold	Rose Geranium	Rose Santa Teresinha
Theoretical ABTS	(mmol Trolox g^−1^ DW)	0.0027	0.0002	0.0007	0.0034
Measured ABTS	(mmol Trolox g^−1^ DW)	0.15	0.14	0.92	0.93
Explained	(%)	1.81	0.17	0.07	0.36
Synergy		56	700	1314	274
Theoretical DPPH	(mmol Trolox g^−1^ DW)	0.0021	0.0002	0.0005	0.003
Measured DPPH	(mmol Trolox g^−1^ DW)	0.2	0.11	0.96	0.87
Explained	(%)	1.11	0.1655	0.0525	0.31
Synergy		95	550	1920	322
Theoretical FRAP	(mmol Trolox g^−1^ DW)	0.0021	0.0002	0.0006	0.003
Measured FRAP	(mmol Trolox g^−1^ DW)	0.13	0.12	0.85	0.78
Explained	(%)	1.58	0.15	0.0655	0.38
Synergy		61.9	600.0	1416.7	269.0

## Data Availability

Data are contained within the article and Supplementary Materials.

## Supplementary Materials

The following supporting information can be downloaded at: https://www.mdpi.com/article/10.3390/antiox14030282/s1, Table S1: Theoretical antioxidant contributions of individual phenolic compounds concentrations determined by high-performance liquid chromatography (HPLC) and literature-derived Trolox equivalent antioxidant capacity (TEAC); Table S2: Theoretical antioxidant contributions of phenolic compounds to ABTS, DPPH, and FRAP activities. Table S3: Variance explained by PCA (Principal Component Analysis); Table S4: PCA loadings for individual phenolic compounds; Table S5: Performance of the Partial Least Squares (PLS) regression model; Table S6: VIP scores for all compounds.

## References

[1] Kumari P., Bhargava B. (2021). Phytochemicals from edible flowers: Opening a new arena for healthy lifestyle. J. Funct. Foods.

[2] Zheng J., Meenu M., Xu B. (2019). A systematic investigation on free phenolic acids and flavonoids profiles of commonly consumed edible flowers in China. J. Pharm. Biomed. Anal..

[3] Gonçalves F., Gonçalves J.C., Ferrão A.C., Correia P., Guiné R.P. (2020). Evaluation of phenolic compounds and antioxidant activity in some edible flowers. Open Agric..

[4] Prabawati N.B., Oktavirina V., Palma M., Setyaningsih W. (2021). Edible flowers: Antioxidant compounds and their functional properties. Horticulturae.

[5] Hegde A.S., Gupta S., Sharma S., Srivatsan V., Kumari P. (2022). Edible rose flowers: A doorway to gastronomic and nutraceutical research. Food Res. Int..

[6] Kandylis P. (2022). Phytochemicals and Antioxidant Properties of Edible Flowers. Appl. Sci..

[7] Barba F.J., Esteve M.J., Frígola A. (2014). Bioactive components from leaf vegetable products. Stud. Nat. Prod. Chem..

[8] Murkovic M., Caballero B., Finglas P.M., Toldrá F. (2016). Phenolic Compounds: Occurrence, Classes, and Analysis. Encyclopedia of Food and Health.

[9] Takahashi J.A., Rezende F.A.G.G., Moura M.A.F., Dominguete L.C.B., Sande D. (2020). Edible flowers: Bioactive profile and its potential to be used in food development. Food Res. Int..

[10] Chensom S., Okumura H., Mishima T. (2019). Primary screening of antioxidant activity, total polyphenol content, carotenoid content, and nutritional composition of 13 edible flowers from Japan. Prev. Nutr. Food Sci..

[11] Varshney A., Dahiya P., Mohan S. (2023). Growth, biochemical, and antioxidant response of pot marigold (*Calendula officinalis* L.) grown in fly ash amended soil. Int. J. Phytoremediation.

[12] Jan N., John R. *Calendula officinalis*: An Important Medicinal Plant with Potential Biological Properties. Proceedings of the Indian National Science Academy.

[13] Ben ElHadj Ali I., Tajini F., Boulila A., Jebri M.A., Boussaid M., Messaoud C., Sebaï H. (2020). Bioactive compounds from Tunisian *Pelargonium graveolens* (L’Hér.) essential oils and extracts: α-amylase and acethylcholinesterase inhibitory and antioxidant, antibacterial and phytotoxic activities. Ind. Crops Prod..

[14] Colling J., Groenewald J.H., Makunga N.P. (2010). Genetic alterations for increased coumarin production lead to metabolic changes in the medicinally important *Pelargonium sidoides* DC (Geraniaceae). Metab. Eng..

[15] El Aanachi S., Gali L., Nacer S.N., Bensouici C., Dari K., Aassila H. (2020). Phenolic contents and in vitro investigation of the antioxidant, enzyme inhibitory, photoprotective, and antimicrobial effects of the organic extracts of *Pelargonium graveolens* growing in Morocco. Biocatal. Agric. Biotechnol..

[16] Gonçalves A.F.K., Friedrich R.B., Boligon A.A., Piana M., Beck R.C.R., Athayde M.L. (2012). Anti-oxidant capacity, total phenolic contents and HPLC determination of rutin in *Viola tricolor* (L) flowers. Free. Radic. Antioxid..

[17] Piana M., Silva M.A., Trevisan G., De Brum T.F., Silva C.R., Boligon A.A., Oliveira S.M., Zadra M., Hoffmeister C., Rossato M.F. (2013). Antiinflammatory effects of *Viola tricolor* gel in a model of sunburn in rats and the gel stability study. J. Ethnopharmacol..

[18] Vukics V., Ringer T., Kery A., Bonn G.K., Guttman A. (2008). Analysis of heartsease (*Viola tricolor* L.) flavonoid glycosides by micro-liquid chromatography coupled to multistage mass spectrometry. J. Chromatogr. A.

[19] Alizadeh Z. (2021). Essential oil, total phenolic, flavonoids, anthocyanins, carotenoids and antioxidant activity of cultivated Damask Rose (*Rosa damascena*) from Iran: With chemotyping approach concerning morphology and composition. Sci. Hortic..

[20] Nowicka P., Wojdyło A. (2019). Anti-hyperglycemic and anticholinergic effects of natural antioxidant contents in edible flowers. Antioxidants.

[21] Peng A., Lin L., Zhao M., Sun B. (2019). Classification of edible chrysanthemums based on phenolic profiles and mechanisms underlying the protective effects of characteristic phenolics on oxidatively damaged erythrocyte. Food Res. Int..

[22] Pires T.C.S.P., Dias M.I., Barros L., Calhelha R.C., Alves M.J., Oliveira M.B.P.P., Santos-Buelga C., Ferreira I.C.F.R. (2018). Edible flowers as sources of phenolic compounds with bioactive potential. Food Res. Int..

[23] de Morais J.S., Sant’Ana A.S., Dantas A.M., Silva B.S., Lima M.S., Borges G.C., Magnani M. (2020). Antioxidant activity and bioaccessibility of phenolic compounds in white, red, blue, purple, yellow and orange edible flowers through a simulated intestinal barrier. Food Res. Int..

[24] Escher G.B., Borges L.D.C.C., Santos J.S., Cruz T.M., Marques M.B., Do Carmo M.A.V., Azevedo L., Furtado M.M., Sant’ana A.S., Wen M. (2019). From the field to the pot: Phytochemical and functional analyses of calendula officinalis l. flower for incorporation in an organic yogurt. Antioxidants.

[25] Shikov V., Kammerer D.R., Mihalev K., Mollov P., Carle R. (2012). Antioxidant capacity and colour stability of texture-improved canned strawberries as affected by the addition of rose (*Rosa damascena* Mill.) petal extracts. Food Res. Int..

[26] Yu M., Gouvinhas I., Rocha J., Barros A.I.R.N.A. (2021). Phytochemical and antioxidant analysis of medicinal and food plants towards bioactive food and pharmaceutical resources. Sci. Rep..

[27] Aires A., Carvalho R. (2020). Kiwi fruit residues from industry processing: Study for a maximum phenolic recovery yield. J. Food Sci. Technol..

[28] Natarajan K., Singh S., Burke T.R., Grunberger D. (1996). Caffeic acid phenethyl ester is a potent and specific inhibitor of activation of nuclear transcription factor NF-κB. Proc. Natl. Acad. Sci. USA.

[29] Sato Y., Shibuya H., Taira N., Kadoma Y. (1996). Inhibitory effect of caffeic acid phenethyl ester on nuclear factor-kappaB activation in human T lymphocytes. J. Immunol..

[30] Van Dijk A.E., Olthof M.R., Meeuwesen S.E., Heine R.J., Van Dam R.M. (2009). The effect of coffee consumption on glucose metabolism: A systematic review and meta-analysis of randomized controlled trials. Am. J. Clin. Nutr..

[31] Sánchez-Ramírez C., González-Romero S.A., Ruiz-Moreno C., Fernández-López J., Pérez-Álvarez J.A. (2013). Influence of the drying method on the antioxidant properties and color of tomato (*Lycopersicon esculentum*). Food Chem..

[32] Sánchez-Moreno C., de Ancos B. (2002). Phenolic acids as potential natural antioxidants in food preservation. Trends Food Sci. Technol..

[33] Boots A.W., Haenen G.R.M.M., Bast A. (2008). Health effects of quercetin: From antioxidant to nutraceutical. Eur. J. Pharmacol..

[34] Middleton E., Kandaswami C., Theoharides T.C. (2000). The effects of plant flavonoids on mammalian cells: Implications for inflammation, heart disease, and cancer. Pharmacol. Rev..

[35] Shi Y., Williamson G. (2016). Quercetin lowers plasma uric acid in pre-hyperuricaemic males: A randomised, double-blinded, placebo-controlled, cross-over trial. Br. J. Nutr..

[36] Joseph J.A., Shukitt-Hale B., Casadesus G. (2005). Reversing the deleterious effects of aging on neuronal communication and behavior: Beneficial properties of fruit polyphenolic compounds. Am. J. Clin. Nutr..

[37] Müller L., Gnoyke S., Popken A.M., Böhm V. (2010). Antioxidant Capacity and Related Parameters of Different Fruit Formulations. Food Sci. Technol..

[38] Cabrera C., Artacho R., Giménez R. (2006). Beneficial effects of green tea—A review. J. Am. Coll. Nutr..

[39] Hodgson J.M., Croft K.D. (2010). Dietary flavonoids: Effects on endothelial function and nitric oxide production. J. Sci. Food Agric..

[40] Rice-Evans C., Miller N.J., Paganga G. (1997). Antioxidant properties of phenolic compounds. Trends Plant Sci..

[41] Schweiggert U., Carle R., Schieber A. (2015). Conventional and alternative processes for spice production—A review. Trends Food Sci. Technol..

[42] Singh B.N., Shankar S., Srivastava R.K. (2016). Green tea catechin, epigallocatechin-3-gallate (EGCG): Mechanisms, perspectives and clinical applications. Biochem. Pharmacol..

[43] González-Barrio R., Periago M.J., Luna-Recio C., Navarro-González I., García-Alonso J. (2010). Chemical and biological properties of marigold (*Tagetes erecta*) extract. Food Res. Int..

[44] Miliauskas G., Venskutonis P.R., van Beek T.A. (2004). Screening of radical scavenging activity of some medicinal and aromatic plant extracts. Food Chem..

[45] Huang D., Ou B., Prior R.L. (2005). The chemistry behind antioxidant capacity assays. J. Agric. Food Chem..

[46] Prior R.L., Wu X., Schaich K. (2005). Standardized methods for the determination of antioxidant capacity and phenolics in foods and dietary supplements. J. Agric. Food Chem..

[47] Moon J.K., Shibamoto T. (2009). Antioxidant assays for plant and food components. J. Agric. Food Chem..

[48] Dai J., Mumper R.J. (2010). Plant phenolics: Extraction, analysis and their antioxidant and anticancer properties. Molecules.

[49] Cao G., Sofic E., Prior R.L. (1997). Antioxidant and prooxidant behavior of flavonoids: Structure-activity relationships. Free Radic. Biol. Med..

[50] Sies H. (1997). Oxidative stress: Oxidants and antioxidants. Exp. Physiol..

[51] Pietta P.G. (2000). Flavonoids as antioxidants. J. Nat. Prod..

[52] Tsao R. (2010). Chemistry and biochemistry of dietary polyphenols. Nutrients.

[53] Gulcin I. (2012). Antioxidant activity of food constituents: An overview. Arch. Toxicol..

[54] Kähkönen M.P., Hopia A.I., Heinonen M. (2001). Berry phenolics and their antioxidant activity. J. Agric. Food Chem..

[55] Balasundram N., Sundram K., Samman S. (2006). Phenolic compounds in plants and agri-industrial by-products: Antioxidant activity, occurrence, and potential uses. Food Chem..

[56] Palafox-Carlos H., Ayala-Zavala J.F., González-Aguilar G.A. (2012). The role of dietary fiber in the bioaccessibility and bioavailability of fruit and vegetable antioxidants. J. Food Sci..

[57] Brand-Williams W., Cuvelier M.E., Berset C. (1995). Use of a free radical method to evaluate antioxidant activity. LWT Food Sci. Technol..

[58] Eriksson L., Johansson E., Kettaneh-Wold N., Trygg J., Wikström C., Wold S. (2006). Multi- and Megavariate Data Analysis: Part I: Basic Principles and Applications.

[59] Wold S., Sjöström M., Eriksson L. (2001). PLS-regression: A basic tool of chemometrics. Chemom. Intell. Lab. Syst..

[60] Roldán E., Sánchez-Moreno C., Ancos B.D., Cano M.P. (2008). Characterization of onion (*Allium cepa* L.) by-products as food ingredients with antioxidant and antibrowning. Prop. Food Chem..

[61] Fernandes G., Silva G., Pavan A., Chiba D., Chin C., Santos J.D. (2017). Epigenetic regulatory mechanisms induced by resveratrol. Nutrients.

[62] Trygg J., Wold S. (2002). Orthogonal Projections to Latent Structures (O-PLS). J. Chemom..

[63] Llorach R., Gil-Izquierdo A., Ferreres F., Tomás-Barberán F.A. (2003). HPLC-DAD-MS/MS ESI Characterization of Unusual Highly Glycosylated Acylated Flavonoids from Cauliflower (*Brassica oleracea* L. var. botrytis) agroindustrial byproducts. J. Agric. Food Chem..

[64] Granato D., Putnik P., Kovačević D.B., Santos J.S., Calado V., Rocha R.S., Cruz A.G.D., Jarvis B., Rodionova O.Y., Pomerantsev A. (2018). Trends in chemometrics: Food authentication, microbiology, and effects of processing. Compr. Rev. Food Sci. Food Saf..

[65] Farah A., Donangelo C.M. (2006). Phenolic compounds in coffee. Braz. J. Plant Physiol..

