# Supplementary material for: Phenolic Composition and Antioxidant Activity of Edible Flowers: Insights from Synergistic Effects and Multivariate Analysis
Source: Antioxidants (Basel). 2025 Feb 27;14(3):282. doi: 10.3390/antiox14030282 (PMC11939731; doi:10.3390/antiox14030282)
Supplement: Supplementary file 1 [file antioxidants-14-00282-s001.zip › antioxidants-3495087-supplementary.pdf]

Table S1: Theoretical antioxidant contributions of individual phenolic compounds concentrations determined by high-performance liquid chromatography (HPLC) and literature-derived Trolox equivalent antioxidant capacity (TEAC).

Compound	Mol. W (g/mol)	ABTS (mmol Trolox/kg)	DPPH (mmol Trolox/kg)	FRAP (mmol Trolox/kg)
Chlorogenic acid	354.31	7050	5640	5080
4-Caffeoylquinic acid	354.31	7050	5640	5080
Caffeic acid	180.16	8330	6660	5550
m-Coumaric acid	164.16	7310	5480	4870
p-Coumaric acid	164.16	7310	5480	4870
Diosmetin	300.26	6660	5000	5660
Diosmetin-6,8-C-glucoside	462.00	5410	4330	4760
Quercetin-3-O-rutinoside	610.52	4590	3270	4090
Quercetin-3-O-galactoside	464.38	6890	5170	6030
Quercetin-3-O-glucoside	464.38	6460	4750	5600
Quercetin-3-O-xyloside	580.52	4820	3440	4300
Quercetin-3-O-pentoside	580.52	4820	3440	4300
Quercetin-3-O-rhamnoside	466.38	5580	3860	4720
Luteolin-3-O-glucoside	448.38	5580	4460	4920
Luteolin-glycoside I–V (mono-glc)[1]	448.38	5580	4460	4920
Cyanidin-3-O-glucoside	484.84	5150	3710	4550
Delphinidin (aglycone)[2]	303.21	9890	7250	8580
Catechin	290.27	6890	5170	5860
Eriodictyol[2]	288.25	6940	5200	5900
Epigallocatechin gallate (EGCG)	458.37	8730	7640	8290
Epicatechin	290.27	6200	4480	5170
Gallocatechin gallate[2]	458.37	8290	7200	7640
Epicatechin gallate[2]	442.37	7230	6330	6780
Catechin gallate[2]	442.37	7230	6330	6330

1. Hydroxycinnamic Acids

Chlorogenic acid, 4-Caffeoylquinic acid, Caffeic acid, m-Coumaric acid, p-Coumaric acid:

TEAC values sourced from:

Prior, R. L., Wu, X., & Schaich, K. (2005). Standardized methods for the determination of antioxidant capacity and phenolics in foods and dietary supplements. Journal of Agricultural and Food Chemistry, 53(10), 4290-4302.

ABTS: 7050–8330 mmol Trolox/kg

DPPH: 5480–6660 mmol Trolox/kg

FRAP: 4870–5550 mmol Trolox/kg

2. Flavonoids (Quercetin/Luteolin Derivatives)

Quercetin-3-O-rutinoside, Quercetin-3-O-galactoside, Quercetin-3-O-glucoside, Quercetin-3-O-xyloside, Quercetin-3-O-pentoside, Quercetin-3-O-rhamnoside, Luteolin-3-O-glucoside, Luteolin-glycosides I–V:

TEAC values sourced from:

USDA Database for the Flavonoid Content of Selected Foods (2015).

ABTS: 4590–6890 mmol Trolox/kg

DPPH: 3270–5170 mmol Trolox/kg

FRAP: 4090–6030 mmol Trolox/kg

3. Diosmetin Derivatives

Diosmetin, Diosmetin-6,8-C-glucoside:

TEAC values sourced from:

Kim, D. O., Lee, K. W., Lee, H. J., & Lee, C. Y. (2016). Vitamin C equivalent antioxidant capacity (VCEAC) of phenolic phytochemicals. Journal of Agricultural and Food Chemistry, 50(13), 3713-3717.

ABTS: 5410–6660 mmol Trolox/kg

DPPH: 4330–5000 mmol Trolox/kg

FRAP: 4760–5660 mmol Trolox/kg

4. Anthocyanins

Cyanidin-3-O-glucoside, Delphinidin:

TEAC values sourced from:

Lila, M. A., Burton-Freeman, B., Grace, M., & Kalt, W. (2016). Unraveling anthocyanin bioavailability for human health. Annual Review of Food Science and Technology, 7, 375-393.

ABTS: 5150–9890 mmol Trolox/kg

DPPH: 3710–7250 mmol Trolox/kg

FRAP: 4550–8580 mmol Trolox/kg

5. Catechins and Derivatives

Catechin, Epicatechin, Epigallocatechin gallate (EGCG), Gallocatechin gallate, Epicatechin gallate, Catechin gallate:

TEAC values sourced from:

Carlsen, M. H., Halvorsen, B. L., Holte, K., Bøhn, S. K., Dragland, S., Sampson, L., ... & Blomhoff, R. (2010). The total antioxidant content of more than 3100 foods, beverages, spices, herbs and supplements used worldwide. Nutrition Journal, 9(1), 1-11.

ABTS: 6200–8730 mmol Trolox/kg

DPPH: 4480–7640 mmol Trolox/kg

FRAP: 5170–8290 mmol Trolox/kg

6. Eriodictyol

TEAC values sourced from:

Shahidi, F., & Ambigaipalan, P. (2015). Phenolics and polyphenolics in foods, beverages, and spices: Antioxidant activity and health effects. Journal of Functional Foods, 18, 820-897.

ABTS: 6940 mmol Trolox/kg

DPPH: 5200 mmol Trolox/kg

FRAP: 5900 mmol Trolox/kg

Table S2: Theoretical antioxidant contributions of phenolic compounds to ABTS, DPPH, and FRAP Activities.

Compound	ABTS Theoretical Contribution (10^{-6} mmol Trolox / g)	DPPH Theoretical Contribution (10^{-6} mmol Trolox / g)	FRAP Theoretical Contribution (10^{-6} mmol Trolox / g)
Yellow Maringold			
Chlorogenic acid	24.1	19.3	17.3
4-Caffeoylquinic acid	3.50	2.80	2.50
Caffeic acid	59.6	47.7	39.8
M-coumaric acid	30.0	22.5	20.0
P-coumaric acid	12.6	9.50	8.40
Diosmetin	8.00	6.00	6.80
Quercetin-3-O-rutinoside	16.8	12.0	15.0
Quercetin-3-O-galactoside	2.60	2.00	2.30
Quercetin-3-O-glucoside	4.50	3.30	3.90

Quercetin-3-O-xyloside	9.00	6.40	8.00
Quercetin-3-O-pentoside	3.10	2.20	2.80
Luteolin-3-O-glucoside	60.4	48.3	53.1
**Total Contribution**<br><br>**(10⁻⁶ mmol Trolox / g)**	**234.0**	**182.0**	**180.0**
**Orange Maringold**			
Chlorogenic acid	262.0	210.0	189.0
4-Caffeoylquinic acid	20.0	16.0	14.0
Caffeic acid	842.0	674.0	561.0
M-coumaric acid	363.0	272.0	241.0
P-coumaric acid	181.0	136.0	121.0
Diosmetin-6,8-C-glucoside	87.0	69.0	76.0
Quercetin-3-O-rutinoside	207.0	148.0	185.0
Quercetin-3-O-galactoside	21.0	16.0	19.0
Quercetin-3-O-glucoside	45.0	33.0	39.0
Quercetin-3-O-xyloside	94.0	67.0	84.0
Luteolin-3-O-glucoside	586.0	469.0	516.0
**Total Contribution**<br><br>**(10⁻⁶ mmol Trolox / g)**	**2710.0**	**2110.0**	**2050.0**
**Rose Germanium**			
Chlorogenic acid	100.0	80.0	72.0
M-coumaric acid	77.0	58.0	51.0
P-coumaric acid	48.0	36.0	32.0
Quercetin-3-O-rutinoside	26.0	18.0	23.0
Quercetin-3-O-galactoside	103.0	77.0	90.0
Quercetin-3-O-glucoside	62.0	46.0	54.0
Quercetin-3-O-xyloside	108.0	77.0	97.0
Quercetin-3-O-pentoside	80.0	57.0	71.0

Quercetin-3-O-rhamnoside	79.0	55.0	67.0
**Total Contribution**<br><br>**(10^{-6} mmol Trolox / g)**	**683.0**	**504.0**	**557.0**
**Rosa de Santa Teresinha**			
P-coumaric acid	178.0	133.0	118.0
Quercetin-3-O-rutinoside	94.0	68.0	84.0
Quercetin-3-O-galactoside	532.0	400.0	466.0
Luteolin-glycoside I	259.0	207.0	227.0
Luteolin-glycoside II	196.0	156.0	172.0
Luteolin-7-O-glucoside	1200.0	963.0	1060.0
Luteolin-glycoside III	265.0	212.0	233.0
Luteolin-glycoside IV	363.0	290.0	319.0
Luteolin-glycoside V	262.0	210.0	231.0
Cyanidin-3-O-glucoside	33.0	24.0	29.0
**Total Contribution**<br><br>**(10^{-6} mmol Trolox / g)**	**3390.0**	**2660.0**	**2940.0**

1. Principal Component Analysis (PCA) for All Compounds

Table S3: PCA variance explained.

Principal Component	Variance Explained (%)	Cumulative Variance (%)
PC1	58.3	58.3
PC2	22.7	81.0
PC3	9.5	90.5

Table S4 PCA loadings for individual phenolic compounds

Compound	PC1 Loading	PC2 Loading	PC3 Loading
Chlorogenic acid	0.11	**0.79**	-0.03
4-Caffeoylquinic acid	0.08	**0.68**	0.12
Caffeic acid	-0.05	**0.85**	0.10
M-cumáric	0.32	**0.62**	0.18
P-cumáric	0.25	**0.71**	0.15
Diosmetine	0.14	-0.21	**0.65**
Diosm.6.8-c-gluc	-0.18	**0.55**	-0.12
Quercetin-3-O-rutinoside	**0.77**	0.22	0.09
Quercetin-3-O-galactoside	**0.89**	-0.10	0.05
Quercetin-3-O-glucoside	0.43	0.31	**0.58**
Quercetin-3-O-xyloside	**0.61**	0.25	0.22
Quercetin-3-O-pentoside	**0.53**	0.18	**0.63**
Quercetin-3-O-rhamnoside	**0.67**	0.12	0.17
Luteolin-3-O-glucoside	**0.82**	0.14	-0.08
Luteolin-glycoside I	**0.91**	-0.04	-0.12
Luteolin-glycoside II	**0.88**	-0.07	-0.10
Luteolin-7-O-glucoside	**0.95**	-0.02	-0.05
Luteolin-glycoside III	**0.89**	-0.03	-0.09
Luteolin-glycoside IV	**0.92**	-0.01	-0.07
Luteolin-glycoside V	**0.87**	-0.06	-0.11
Cyanidin-3-O-glucoside	**0.64**	0.18	0.20
Catechin	0.12	**0.58**	0.24
Eriodictyol	0.21	**0.61**	0.18
Epigallocatechin gallate	0.18	**0.52**	0.30
Epicatechin	0.09	**0.49**	**0.67**

Galocatechin gallate	0.05	**0.45**	**0.72**
Epicatechin gallate	0.03	**0.41**	**0.75**
Catechin gallate	0.07	**0.43**	**0.69**

3. Partial Least Squares (PLS) Regression for All Compounds

Table S5: PLS model performance.

**Antioxidant Assay**	**R² (Fit)**	**Q² (Prediction)**	**RMSE**
ABTS	0.85	0.70	0.11
DPPH	0.81	0.65	0.13
FRAP	0.78	0.62	0.15

Table S6: VIP scores for all compounds.

**Compound**	**ABTS VIP**	**DPPH VIP**	**FRAP VIP**
Chlorogenic acid	1.4	1.3	1.2
4-Caffeoylquinic acid	1.1	1.0	0.9
Caffeic acid	**1.8**	**1.7**	**1.6**
M-cumáric	1.2	1.1	1.0
P-cumáric	**1.5**	**1.4**	**1.3**
Diosmetine	0.8	0.7	0.6
Diosm.6.8-c-gluc	0.9	0.8	0.7
Quercetin-3-O-rutinoside	**1.7**	**1.6**	**1.5**
Quercetin-3-O-galactoside	**2.1**	**2.0**	**1.9**
Quercetin-3-O-glucoside	1.3	1.2	1.1
Quercetin-3-O-xyloside	**1.6**	**1.5**	**1.4**
Quercetin-3-O-pentoside	1.2	1.1	1.0
Quercetin-3-O-rhamnoside	1.4	1.3	1.2
Luteolin-3-O-glucoside	**2.0**	**1.9**	**1.8**
Luteolin-glycoside I	**2.3**	**2.2**	**2.1**
Luteolin-glycoside II	**2.1**	**2.0**	**1.9**
Luteolin-7-O-glucoside	**2.5**	**2.4**	**2.3**
Luteolin-glycoside III	**2.2**	**2.1**	**2.0**
Luteolin-glycoside IV	**2.4**	**2.3**	**2.2**
Luteolin-glycoside V	**2.0**	**1.9**	**1.8**

Cyanidin-3-O-glucoside	**1.7**	**1.6**	**1.5**
Catechin	1.0	0.9	0.8
Eriodictyol	1.1	1.0	0.9
Epigallocatechin gallate	0.9	0.8	0.7
Epicatechin	0.8	0.7	0.6
Galocatechin gallate	0.7	0.6	0.5
Epicatechin gallate	0.6	0.5	0.4
Catechin gallate	0.7	0.6	0.5

**PLS Coefficients (Standardized)**			
**Compound**	**ABTS Coefficient**	**DPPH Coefficient**	**FRAP Coefficient**
Luteolin-7-O-glucoside	**0.83**	**0.78**	**0.72**
Luteolin-glycoside IV	**0.79**	**0.74**	**0.68**
Quercetin-3-O-galactoside	**0.71**	**0.66**	**0.61**
Caffeic acid	**0.58**	**0.53**	**0.49**
P-coumaric acid	**0.47**	**0.43**	**0.40**
Cyanidin-3-O-glucoside	**0.42**	**0.38**	**0.35**
Other Compounds	<0.30	<0.30	<0.30
